# Physiological response, testicular function, and health indices of rabbit males fed diets containing phytochemicals extract under heat stress conditions

**DOI:** 10.5455/javar.2021.h510

**Published:** 2021-06-19

**Authors:** Khaled Hassan El-Kholy, Wael Mohamed Wafa, Hamdy Abdala El-Nagar, Abdelrhman Mosad Aboelmagd, Ibrahim Talat El-Ratel

**Affiliations:** 1 Department of Poultry Production, Faculty of Agriculture, Damietta University, Damietta 34517, Egypt; 2 Department of Cattle Breeding Research, Agricultural Research Center, Animal Production Research Institute, Giza 12618, Egypt; 3 Department of Biotechnology Research, Agricultural Research Center, Animal Production Research Institute, Giza 12618, Egypt

**Keywords:** Heat stress, phytochemicals, rabbit males, testicular functions

## Abstract

**Objective:**

The present study aimed to evaluate the impact of dietary turmeric or garlic extract on physiological responses, hematological parameters, antioxidants status, testicular functions, and health indices of heat-stressed rabbit males.

**Materials and Methods:**

Seventy bucks were distributed into seven experimental groups (ten/group). A commercial diet (CD) was fed to rabbit males in the control group, while males in G2, G3, and G4 were fed CD with turmeric at 30, 60, and 90 mg/kg, respectively. Males in G5, G6, and G7 were fed CD with garlic extract at 50, 75, and 100 mg/kg, respectively, for 8 weeks.

**Results:**

The results showed that turmeric or garlic extract levels decreased ear temperature, respiration rate, germ cell apoptotic number, total cholesterol, triglycerides, malondialdehyde level, *libido*, and sperm of dead and abnormal tail (*p* < 0.05). The hemoglobin and red and white blood cells, platelets, total antioxidants capacity, mass motility, mitochondrial potential, normal, acrosome reacted, normality, and a total functional fraction of spermatozoa and relative of epididymal weight were increased (*p* < 0.05). The increased packed cell volume percentage and initial semen fructose were significant only when 100 mg per kg garlic extract was supplemented.

**Conclusion:**

Phytochemicals extract supplementation can be an effective dietary supplementation to eliminate heat stress and improve health, oxidative capacity, and testicular functions of rabbit males.

## Introduction

High environmental temperature changes farm animals’ health status and reproductive efficiency by causing heat stress (HS). The HS causes alterations in physiology, behavior, and production in animals [1,2]. Oxidative stress is one of the main physiological alterations in rabbits under HS due to increased reactive oxygen species (ROS) and imbalances in antioxidant enzyme production [3]. These conditions can impair the health status by disturbance of the physiological homeostasis and marked reduction in the reproductive performance of male rabbits [2]. Negative influences on the testis structure and functions may also cause DNA damage and low fertilizing capacity [3]. Several authors have reported deterioration in males’ general health and testicular functions because of prolonged intense HS in the tropical and sub-tropical regions [2,4,5]. Thus, focusing on maintaining reproductive efficacy and health status in rabbit breeding farms under HS has become necessary over the years. It is hypothesized that the addition of phytochemicals to the diets was found to ameliorate and protect the animals from the impaired effects on spermatogenesis under HS [2,4,6].

Turmeric (*Curcuma longa*), one of such phytochemicals, is a member of the *Zingiberaceae* family [7]. Also known for its therapeutic and physiological activities, turmeric contained essential oils, including some critical trace mineral elements [8]. Curcuminoids and their derivatives in turmeric have biological activities and pharmacological properties [9]. Studies have reported that turmeric has different effects, such as antioxidant, anticancer, anti-inflammatory, and anti-cardiovascular disease [10]. Turmeric also protects DNA damage, prevents the spermatogenic cells from apoptosis, and develops the testicular tissues [11]. In male rats, turmeric might be promising in enhancing semen quality [12]. In roosters, dietary curcumin at 30 mg per bird improved semen quality and fertility [13].

Garlic (*Allium sativum *L*.*), as a medicinal and therapeutic agent [14], contains flavonoids and sulfur-containing compounds as antioxidants [15,16]. These compounds act against tumor, hyperlipidemia, hypocholesterolemia, and oxidant [17].

Under normal conditions, garlic with vitamin-E supplementation increased spermatogenesis and semen characteristics in males [18]. Also, dietary garlic increases testosterone levels in male rats fed a high fatty diet [19]. In Koekoek breeder cocks, dietary garlic supplementation can enhance reproductive efficiency [20]. Results reported on the effects of various levels of garlic or turmeric on rabbit male performance are conflicted. Dietary supplementation of garlic (at 2, 4, and 6 gm/kg) or turmeric (at 2, 4, and 6 gm/kg) was reported to improve the antioxidant capacity of the liver in growing rabbits [14]. However, the information on the optimal level of garlic or turmeric extracts, as natural antioxidants, was not studied for rabbit males under HS. It was hypothesized that phytochemical extracts could be an economical and safe strategy to improve rabbit males’ health status and testicular functions under HS conditions. It is well known that the levels of turmeric or garlic extract play a key role in determining which effect its administration would have on the physiology and biochemistry of the body organs. Therefore, the current study aimed to evaluate the impact of dietary supplementation of turmeric at 30, 60, and 90 mg/kg, or garlic extract at 50, 75, and 100 mg/kg on physiological responses and hematological parameters, antioxidants status, testicular functions, and health indices of rabbit males under HS conditions.

## Materials and Methods

### Ethical approval

The current study protocol used in this study was approved by the Animal Care and Ethics committee of the Department of Poultry Production, Faculty of Agriculture, Damietta University, Egypt (Approval number: 03/2018/du.edu).

### Phytochemicals supplement, and analysis

Samples (*n* = 3) from turmeric and garlic powder were taken for assaying the concentration of mono-unsaturated fatty acids (MUFA) and poly-unsaturated fatty acids (PUFA), saturated fatty acids (SFA), and unsaturated fatty acids (UFA) according to the methods described by Radwan et al. [21]. Also, antioxidant activity inhibition percentage [22], total phenolic contents [23], and total flavonoids [24] were determined. Contents of mineral [25] and vitamin A, C, and D [26,27] were determined.

### Animals, management, and experimental design

Seventy sexually mature Animal Production Research Institute (APRI) rabbit males, 6–7month-old, were used in the present study [28]. Individual housing in galvanized enclosures (40 × 50 × 35 cm^3^) was used for males with feeders containing food and water in automatic stainless-steel nipple drinkers under similar management and environmental conditions hygienic factors. At the beginning of the experiment, the rabbits were allowed to feed the experimental diets for 1 week as an adaptation interval. The same diet was formulated to enable nutritional needs [29]. Ingredients and chemical analyses of the basal diet [30] are presented in Table 1. Food and fresh, clean water were offered *ad libitum*. The experimental males were divided into seven experimental groups (10 in each). Seventy males were distributed into seven experimental groups (10/group). A commercial diet (CD) was fed to rabbit males in the control group, while males in G2, G3, and G4 were fed CD with turmeric at 30, 60, and 90 mg/kg, respectively. Males in G5, G6, and G7 were fed CD with garlic extract at 50, 75, and 100 mg/kg, respectively, for 8 weeks as a treatment period, followed by 8 weeks for semen collection. Contents of fatty acids, antioxidants indices, minerals, and vitamins of turmeric and garlic powder are shown in Table 2.

### Climatic parameters

During an adaptation and the experimental periods, the recorded maximal and minimal ambient temperatures inside the rabbitry were 30.45°C ± 0.32°C and 26.24°C ± 0.51°C, respectively. However, the relative humidity was 75.35% ± 0.64% and 52.10% ± 1.63%. The calculated values of THI were 29.23 ± 0.30 and 24.57 ± 0.91, indicating severe HS conditions for males in this study [31].

### Physiological parameters

We recorded the weekly body weight (gm) and feed intake (gm/male). We also individually measured ear temperature (ET, °C) by placing the probe of a digital thermometer (Type “K” thermocouple, ± 0.01°C, Digi-Sense^®^ Temp Series Thermocouple Thermometers) in direct contact with the internal central area of the auricle. In addition, we recorded the respiration rate (RR, breaths/min) using a hand counter and stopwatch to determine the frequency of the flank movement per minute. The ET and RR were recorded at the same time of measuring ambient temperatures and relative humidity.

**Table 1.** Ingredients and chemical analysis of the basal diet used in different experimental treatments.

Ingredients	%	Chemical analysis	%
Clover hay	39.00	Organic matter	90.80
Barley	14.00	Crude protein	17.70
Wheat bran	15.00	Crude fiber	12.40
Soybean meal (44% CP)	17.50	Ether extract	2.27
Maize	9.50	NFE	58.43
Molasses	3.00	Ash	9.20
Di-calcium phosphate	0.50	Digestible energy (Kcal/kg diet)	2670
Limestone	0.80	*The vitamin premix provided the following (per kg of diet):Vitamin A, 6000 IU; Vitamin D_3_, 900 IU; Vitamin E, 40 mg; Vitamin K_3_, 2 mg; Vitamin B_1_, 2 mg; Vitamin B_2_, 4 mg; Vitamin B_6_, 2 mg; Pantothenic acid, 10 mg; Vitamin B_12_, 0.01 mg; Niacin, 50 mg; Folic acid, 3 mg; Biotin, 0.05 mg; Choline, 250 mg; Fe, 50 mg; Mn, 85 mg; Cu, 5 mg; Co, 0.1 mg; Se, 0,1 mg; I, 0.2 mg and Zn, 50 mg.	
Sodium chloride (NaCl)	0.30		
Vitamin & Mineral Mixture*	0.30		
DL-Methionine	0.1		
Clover hay	39.00		
Total	100		

**Table 2.** Contents of fatty acids, antioxidants indices, vitamins, and minerals of turmeric and garlic powder.

Samples		Turmeric	Garlic
Fatty acid (%)			
SFA		12.67 ± 0.96	19.20 ± 1.01
MUFA		69.38 ± 0.55	70.10 ± 0.62
PUFA		18.22 ± 0.66	18.95 ± 0.92
UFA		78.95 ± 2.29	70.1 ± 2.20
Antioxidants indices			
Antioxidant activity inhibition (%)		55.72 ± 1.50	68.20 ± 1.65
Total polyphenols (mg gallic acid equivalent/gm)		1.65 ± 0.08	4.36 ± 0.36
Total flavonoids (mg quercetin equivalent/ /gm)		1.20 ± 0.50	3.25 ± 0.16
Minerals (mg/100 gm)			
Potassium		3.95 ± 0.28	12.18 ± 0.39
Calcium		15.52 ± 1.11	24.93 ± 0.65
Sodium		0.58 ± 0.01	3.98 ± 0.23
Phosphorus		6.58 ± 0.48	11.20 ± 1.14
Iron		3.85 ± 0.19	7.28 ± 2.01
Magnesium		0.90 ± 0.08	1.55 ± 0.25
Zinc		18.20 ± 0.16	8.62 ± 0.23
Vitamins (mg/gm)			
A		2.85 ± 0.39	4.29 ± 0.85
C		0.80 ± 0.04	1.95 ± 0.06
E		0.45 ± 0.01	1.50 ± 0.13

### Semen collection and evaluation

**Figure 1.**
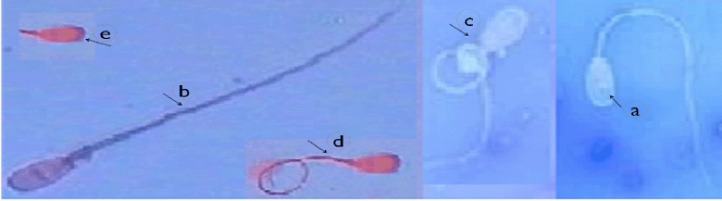
A photomicrograph showing normal (a), dead (b) and tail (c and d) and head (e) abnormalities of sperm in rabbit buck stained with eosin (5%) and nigrosine (10%).

At the termination of the treatment period, semen ejaculates were taken for eight consecutive weeks from 10 males per group (2 times/week), as a collection period (160 ejaculates/group) in the early morning (8 a.m.). The insertion of a rabbit doe into a rabbit male cage until complete ejaculation (reaction time) was recorded on the day of semen collection. Net semen samples after removing the gel in a water bath (37°C) were transferred to the laboratory; then, semen was evaluated. Mass motility (score, 1–5), and percentages of dead (stained sperm), normal, tail abnormal (coiled tail, bent tail, and swollen tail), and head abnormal were determined in each ejaculate (Fig. 1). Percentages of mitochondrial potential and acrosome reactions of spermatozoa were determined [32]. Total functional sperm fraction was calculated according to Abdel-Azeem et al. [33]. Initial fructose concentration immediately after the collection was determined in raw semen [34].

### Blood constituents

In the last week of the experimental period, blood samples (5 ml/male) were taken from the marginal ear vein of 5 males per group before feeding. Each sample was divided into two tubes; the first tube was heparinized, and the second was non-heparinized. The heparinized blood samples were used to test hematological parameters, including hemoglobin concentration (Hb, mg/dl), and count of red blood cells (RBCs, 10^6^/mm^3^), white blood cells (WBCs, 10^3^/mm^3^), platelets, and packed cell volume (PCV, %) using blood hematology analyzer (HB 7021). Non-heparinized blood samples were immediately placed and left for 2–3 h to coagulate, and then clear serum was stored by centrifugation at 700 × g for 20 min and stored at −20°C in 1.5 ml Eppendorf tubes until analysis. Serum total cholesterol (mg/dl) and triglycerides (mg/dl) concentrations were assayed by spectrophotometer (Shimadzu, Kyoto, Japan) and commercial kits (Bio-diagnostics, Giza, Egypt). Serum total antioxidant capacity (TAC) was measured using a commercially available kit (Bio-diagnostics, Giza, Egypt), as previously described [35]. Briefly, the most potent radical, hydroxyl radical, is produced. Firstly, the hydrogen peroxide is mixed with a ferrous ion solution. The sequentially produced radicals (brown colored dianisidinyl radical cations) produced by the hydroxyl radical are potent radicals, then the antioxidative effect of the sample against the potent free radical reactions was determined. The lead concentration was observed by atomic absorption spectrophotometry. A commercially available kit (Bio-diagnostics, Giza, Egypt) was used for serum malondialdehyde (MDA) by Thiobarbituric Acid (TBA) assay [36]. A spectrophotometer (Shimadzu UV-1601, Kyoto, Japan) was used to observe the MDA-TBA complex at 532 nm wavelength.

### Organs weights and health indices

At the end of the experiment, five rabbit males from each were weighed and slaughtered. After slaughtering, testes were isolated immediately from the carcass, trimmed of adhering tissues, and weighed. Also, epididymis was removed from each testis and weighed. Then weights of the testes and epididymal tissues relative body weight were calculated. Testicular measurements (length, width, and thickness) were estimated, and liver, kidney, spleen, and abdominal fat weights were determined, and then organ indices were computed. Evaluation of germ cell apoptosis per seminiferous tubule was performed [5].

### Statistical analysis

The complete randomized design was done for analyzing the obtained data using SAS software (version 9.4). The statistical model *Y*_*ij *_*= μ + G*_*i*_* + e*_*ij*_*. *was used in one-way analysis of variance, where: *Y*_*ij*_ = observed values, *μ* = mean, *G*_*i*_ = effect of treatment, and *e*_*ij*_ = random error. Before the statistical analysis, all percentages were subjected to logarithmic transformation (*log*^10 *x+1*^) to normalize data distribution. The significant differences were set by Duncan’s new multiple range test [37].

## Results

### Physiological parameters

The mean final body weight and feed intake of males were not affected by dietary turmeric or garlic extract supplementation, but the mean respiration rate and ear temperature were reduced (*p* < 0.05) by all treatments compared with the control (Table 3).

### Hematological parameters

The effects of different dietary supplementations on hematological parameters are shown in Table 4. The Hb concentration and count of RBCs and WBCs and platelets were increased (*p* < 0.05) due to dietary supplements; however, the increase in PCV percentage was significant when 100 mg per kg garlic extract was supplemented.

### Serum antioxidants status and lipid profile 

Table 5 shows that blood serum TAC activity increased (*p* < 0.05) due to different supplements while decreasing (*p* < 0.05) serum MDA, total cholesterol, and triglycerides levels.

### Libido and semen characteristics

Results in Table 6 and Table 7 show that all treatments significantly (*p* < 0.05) increased mass motility score and percentages of a normal and total functional fraction of sperm and initial semen fructose (*p* < 0.05). However, the reaction time showed an opposite change and percentages of dead and sperm tail abnormality. The differences in *libido* and abnormal tail sperm percentage among turmeric or garlic extract levels were not significant. However, the increase in initial semen fructose was substantial only when 100 mg per kg garlic extract was supplemented. On the other hand, supplementation of turmeric or garlic extract had no significant effect on abnormal head sperm percentage.

**Table 3.** Impact of phytochemicals extract supplementations on physiological parameters of rabbit males (*n* =10 males per each group).

Item	Control	Turmeric (mg per kg diet)			Garlic extract (mg per kg diet)			*p*-value
		30	60	90	50	75	100	
Final body weight (gm)	3,062.6 ± 101.33	3,067 ± 102.11	3,061.9 ± 102.73	3,063.0 ± 103.02	3,066.1 ± 107.30	3,060.3 ± 104.18	3,065.2 ± 100.61	0.9997
Feed intake (gm/day)	100.40 ± 1.19	102.5 ± 1.83	102.80 ± 1.92	105.90 ± 1.72	107.100 ± 1.53	108.90 ± 1.52	110.40 ± 1.71	0.1031
Respiration rate (Breath/min)	97.40^a^ ± 0.51	94.80 ± 0.71	94.50^b^ ± 0.97	94.45^b^ ± 0.81	94.38^b^ ± 0.89	94.10^b^ ± 0.84	94.39^b^ ± 0.85	0.0001
Ear temperature (°C)	38.89^a^ ± 0.06	38.61 ± 0.09	38.59^b^ ± 0.03	38.52^b^ ± 0.04	38.50^b^ ± 0.15	38.47^b^ ± 0.05	38.45^b^ ± 0.06	0.0010

a,b Means in the same row without common superscript differ significantly (*p* < 0.05).

**Table 4.** Impact of phytochemicals extract supplementations on hematological parameters of rabbit males (*n* = 5 samples per each group).

Item	Control	Turmeric (mg per kg diet)			Garlic extract (mg per kg diet)			*p*-value
		30	60	90	50	75	100	
Hemoglobin (mg/dl)	9.74^b^ ± 0.08	10.36^a^ ± 0.16	10.50^a^ ± 0.18	10.38^a^ ± 0.17	10.54^a^ ± 0.20	10.55^a^ ± 0.17	10.77^a^ ± 0.13	0.0051
Red blood cells (×10^6^/mm^3^)	5.14^d^ ± 0.04	6.06^c^ ± 0.05	6.20^abc^ ± 0.03	6.10^bc^ ± 0.06	6.20^abc^ ± 1.74	6.22^ab^ ± 0.05	6.26^a^ ± 0.04	<0.0001
White blood cells (×10^6^/mm^3^)	7.12^c^ ± 0.05	7.61^a^ ± 0.08	7.40^b^ ± 0.07	7.41^b^ ± 0.02	7.59^a^ ± 0.10	7.59^a^ ± 0.04	7.69^a^ ± 0.03	<0.0001
Platelets (×10^3^/mm^3^)	242.00^b^ ± 2.46	270.20^a^ ± 7.97	274.00^a^ ± 7.64	270.40^a^ ± 11.81	273.40^a^ ± 4.76	271.60 ^a^ ± 4.31	280.40^a^ ± 4.26	0.0140
Packed cell volume (%)	30.80^b^ ± 1.24	33.20^ab^ ± 1.46	35.40^ab^ ± 1.02	33.40^ab^ ± 1.88	33.80^ab^ ± 1.74	34.60^ab^ ± 1.41	36.20^a^ ± 1.50	0.2637

a,b Means in the same row without common superscript differ significantly (*p* < 0.05).

**Table 5.** Impact of phytochemicals extract supplementations on serum antioxidant activity and lipid profile of rabbit males (*n* = 5 samples per each group).

Item	Control	Turmeric (mg per kg diet)			Garlic extract (mg per kg diet)			*p*-value
		30	60	90	50	75	100	
Total antioxidant capacity (mmol/l)	01.05^b^ ± 0.05	01.35^a ^± 0.02	01.41^a^ ± 0.04	01.36^a^ ± 0.02	01.37^a^ ± 0.03	01.36^a^ ± 0.04	01.47^a^ ± 0.05	<0.0001
Malondialdehyde (nmol/l)	36.20^a^ ± 1.8	28.20^b ^± 2.22	24.80^bc^ ± 1.98	28.50^b^ ± 2.33	25.44^bc^ ± 2.70	25.20^bc^ ± 2.48	21.00^c^ ± 1.18	0.0016
Total cholesterol (mg/dl)	116.40^a^ ± 2.73	97.60^b^ ± 4.03	92.40^b^ ± 2.50	97.00^b^ ± 5.12	88.00^b^ ± 2.55	89.60^b^ ± 2.04	92.20^b^ ± 2.69	<.0001
Triglyceride (mg/dl)	163.00^a^ ± 6.04	150.40^ab^ ± 4.96	145.00^b^ ± 3.53	148.00^b^ ± 1.84	140.40^b^ ± 3.70	149.40^ab^ ± 4.68	143.80^b^ ± 3.40	0.0150

a,b,c Means in the same row without common superscript differ significantly (*p* < 0.05).

**Table 6.** Impact of phytochemicals extract supplementations on reaction time and semen characteristics of rabbit males (*n* = 10 males per each group).

Item	Control	Turmeric (mg per kg diet)			Garlic extract (mg per kg diet)			*p*-value
		30	60	90	50	75	100	
*Libido* (sec)	2.01^a^ ± 0.06	1.56^b^ ± 0.05	1.49^b^ ± 0.08	1.53^b^ ± 0.09	1.52^b^ ± 0.05	1.52^b^ ± 0.06	1.54^b^ ± 0.04	0.0001
Mass motility (score 1–5)	3.00^d^ ± 0.25	3.50^abcd^ ± 0.23	3.40^bcd^ ± 0.16	3.22^c^ ± 0.33	3.90^abc^ ± 0.22	4.10^ab^ ± 0.18	4.20^a^ ± 0.25	0.0043
Dead sperm (%)	38.80^a^ ± 1.75	13.10^cd^ ± 1.50	18.40^b^ ± 1.51	17.11^bc^ ± 1.14	11.90^d^ ± 1.52	17.10^bc^ ± 1.37	14.00^bcd^ ± 2.14	<0.0001
Normal sperm (%)	61.20^d^ ± 1.76	86.90^abc^ ± 1.51	81.60^c^ ± 1.51	86.00^ab^ ± 1.14	88.10^a^ ± 1.52	82.90^bc^ ± 1.37	82.889^bc^ ± 1.37	<0.0001
Tail abnormality (%)	16.90^a^ ± 1.11	10.80^b^ ± 0.81	12.50^b^ ± 0.64	12.56^b^ ± 0.78	10.10^b ± ^0.96	11.20^b^ ± 0.90	10.50^b^ ± 0.54	<0.0001
Head abnormality (%)	4.60 ± 0.62	3.40 ± 0.34	3.20 ± 0.39	3.00 ± 0.50	3.30 ± 0.42	3.10 ± 0.35	3.70 ± 0.47	0.2237
TFSF (10^6^/ejaculate)	49.93^d^ ± 1.93	116.29^bc^ ± 3.50	127.84^b^ ± 2.93	114.56^c^ ± 4.99	126.68^bc^ ± 4.22	125.69^bc^ ± 4.47	146.31^a^ ± 6.05	<0.0001
MPS (%)	38.40^b^ ± 0.65	50.80^a^ ± 1.02	50.20^a^ ± 1.41	52.40^a^ ± 0.09	53.20^a^ ± 1.13	51.80^a^ ± 0.52	54.40^a^ ± 1.10	<0.0001
Acrosome reacted sperm (%)	7.60^b^ ± 1.14	15.20^a^ ± 1.20	13.60^a^ ± 0.85	14.80^a^ ± 1.28	16.20^a^ ± 1.25	15.60^a^ ± 1.04	15.0^a^ ± 1.45	0.0006
Initial semen fructose (mg/dl)	70.70^b^ ± 2.56	81.10^a^ ± 3.98	86.90^a^ ± 2.51	86.89^a^ ± 2.53	87.50^a^ ± 1.86	88.20^a^ ± 2.09	86.50^a^ ± 1.61	<0.0001

TFSF = Total functional sperm fraction; MPS = Mitochondrial potential sperm.

a,b,c,d Means in the same row without common superscript differ significantly (*p* < 0.05).

**Table 7.** Impact of phytochemicals extract supplementations on relative organs weights, apoptosis of germ cells and health indices of rabbit males (*n* = 5 males per each group).

Item	Control	Turmeric (mg per kg diet)			Garlic extract (mg per kg diet)			*p*-value
		30	60	90	50	75	100	
Relative testicular weight/kg BW (%)	0.263 ± 0.011	0.287 ± 0.020	0.296 ± 0.015	0.278 ± 0.022	0.294 ± 0.016	0.287 ± 0.0145	0.309 ± 0.012	0.5670
Testicular length (cm)	2.38 ± 0.01	2.39 ± 0.02	2.41 ± 0.01	2.35 ± 0.03	2.39 ± 0.01	2.37 ± 0.02	2.42 ± 0.01	0.8224
Testicular width (cm)	1.25 ± 0.02	1.27 ± 0.01	1.24 ± 0.03	1.25 ± 0.02	1.27 ± 0.01	1.25 ± 0.03	1.28 ± 0.01	0.8057
Testicular thickness (cm)	0.87 ± 0.03	0.90 ± 0.02	0.92 ± 0.01	0.89 ± 0.02	0.90 ± 0.02	0.89 ± 0.01	0.92 ± 0.01	0.3758
Relative epididymal weight/kg BW (%)	0.094^c^ ± 0.003	0.103^ b^ ± 0.001	0.107^ab^ ± 0.002	0.102^b^ ± 0.002	0.106 ± 0.0004	0.105^ab^ ± 0.0011	0.111^a^ ± 0.0015	<0.0001
Germ cell apoptotic/seminiferous tubule (*n*)	49.20^a^ ± 1.38	32.0^b^ ± 1.25	31.6^ b^ ± 1.45	36.80^b^ ± 157	30.2^b^ ± 1.33	31.60^b^ ± 1.47	29.80^b ± ^1.60	<0.0001
Hepato-somatic index	2.66 ± 0.06	2.64 ± 0.09	2.65 ± 0.06	2.648 ± 0.07	2.677 ± 0.08	2.684 ± 0.06	2.681 ± 0.11	0.9995
Renal-somatic index	0.69 ± 0.04	0.68 ± 0.05	0.67 ± 0.06	0.67 ± 0.04	0.724 ± 0.08	0.689 ± 0.041	0.699 ± 0.04	0.9969
Spleen-somatic index	0.055 ± 0.001	0.057 ± 0.00	0.059 ± 0.002	0.057 ± 0.008	0.057 ± 0.001	0.058 ± 0.002	0.059 ± 0.001	0.3604
Relative weight of abdominal fat/kg (%)	2.63^a^ ± 0.09	2.20^b^ ± 0.09	2.07^b^ ± 0.11	2.21^bc^ ± 0.05	2.15^bc^ ± 0.04	2.11^bc^ ± 0.05	1.95^c^ ± 0.06	<0.0001

a,b,c Means in the same row without common superscript differ significantly (*p* < 0.05).

### Testicular and epididymal characteristics and health indices

Relative epididymal weight only was increased (*p* < 0.05) while the number of germ cell apoptotic/seminiferous tubule and abdominal fat weight relative to body weight was decreased (*p* < 0.05) in groups that received turmeric or garlic extract in comparison with the controls. Relative testicular weight and testicular measurements (length, width, and thickness) and hepato-somatic, renal-somatic, and spleen-somatic indexes were not affected by treatment.

## Discussion

Based on the obtained results in our study, the addition of phytochemicals extract of turmeric and garlic provision sustained testicular measurements and health status in rabbit males exposed to HS conditions in terms of maintenance of sperm function and reducing apoptosis of the germ cells via reduction of the oxidative stress. These features are indices of counter the negative effect of HS on health status, characteristics of the testes, and semen quality to detect the positive impacts of phytochemicals extract and minimize HS effect augmentation on the reproductive efficiency of rabbit males. From previous studies, it seems that HS could have negative effects on rabbit farms in hot regions [2,4,6]. In our investigation, the average of THI values (29.23 ± 0.30) cleared that rabbit males were kept under severe HS [31].

Rabbits had no functional sweat glands to increase heat loss. Thus, they have increased ear temperature and respiration rate under HS conditions. All treatments showed a marked reduction of ear temperature and respiration rate compared with the control males. This may indicate the ability of phytochemicals to regulate body temperature [6]. In agreement with our results, El-Desoky et al. [4] reported that vitamins in phytochemicals extract, as antioxidants, have a positive impact on alleviating heat load in rabbits. The phytochemical compounds present in turmeric and garlic extract (Table 2) may facilitate the animal’s ability to regulate body temperature to maintain body homeostasis via provoking the cellular endogenous defense systems, which can cope with the oxidative stress of HS condition [38].

Hematological parameters and antioxidants are usually related to animal physiology and health and evaluate the effect of dietary supplementation [14]. The results indicated positive effects of turmeric and garlic on the hematological parameters. In similar line with the present results, garlic supplementation increased the counts of RBCs and WBCs, and Hb concentration in rabbits [39,40]. The garlic contains natural sulfur compounds which act as antioxidant active substances that imply the antioxidant action of garlic sulfhydryl groups on RBCs counts [39]. Also, vitamins in garlic have a role in RBC formation, maturation, and Hb biosynthesis, absorption, and utilization [39]. The chemical components of garlic seem to act as an active oxygen scavenger that competes with Hb in the RBCs for O_2_ resulting in tissue hypoxia, stimulating the kidney to form and secrete erythropoietin. The Hb synthesis and RBC production were step up by the indirect effect of the end-product of garlic metabolism in the body [40].

Garlic might help in increasing WBC count, consequently the immune system in rabbit [41]. In broilers, dietary turmeric supplementation increased Hb and PCV, enhancing the health status [42], which may be due to the antioxidant activity of turmeric and its effect on strengthening the digestive tract that may improve absorption of iron [42]. Also, some garlic constituents such as flavonoids, steroidal glycosides, alkaloids, saponins, tannins, phenolics, pectin, and amino acids, may play physiological action to stimulate the immunity and the organ function (thymus, spleen, and bone marrow) related to blood cell formation to stimulate more blood production [39,41]. In addition, Alagawany et al. [14] suggested that garlic compounds might have a stimulatory effect on some hematopoietic growth factors (cytokines) that interact with specific receptors on the surface of hematopoietic cells, regulate progenitor cells proliferation and differentiation, and mature cell maturation and function.

It is well-known that the level of MDA increased and TAC level decreased in animals under HS conditions. Lipid peroxidation resulting in oxidative stress was caused by the reaction of ROS with UFA in the cell membranes. In the present study, phytochemicals had more excellent oxidative stability via the increased TAC level and decreased MDA in the blood serum of males under HS conditions. The effectiveness of turmeric or garlic extract could be related to their antioxidant components, which have properties against oxidation, bacteria, fungi, protozoa, and inflammatory [17,42]. The present data indicated that garlic extract had a protective role against HS by improving the health condition, which is probably through its excellent antioxidant properties.

In this way, turmeric or garlic extract eliminates the impaired ROS effects in biological cells [43]. Each extract may be essential for normalizing stability and antioxidant enzyme function [44]. In rabbits, supplementation of antioxidants increases antioxidant enzyme activity and decreases MDA level in blood serum [45,46]. Garlic phytochemicals extract augments antioxidant enzymes and prevents the damage effects of ROS [12]. Elevated levels of antioxidant enzymes may enhance the antioxidant defense system steady-state in rabbit males. Allicin and selenium as garlic components can attenuate the ROS signaling pathways and alleviate the activity of endogenous antioxidant enzymes [47,48].

Interestingly, the increased TAC and reduced lipid peroxidation in rabbit males treated with phytochemicals extract are paralleled with improved *libido* and semen characteristics, which are essential for sperm fertilizability. Enhancement in these parameters was explained to be due to antioxidant components (Table 2), which may prevent cellular damage by increasing the enzymes of the antioxidant defense system of spermatozoa. Reducing ROS generation from lipid peroxidation improves mitochondria function in sperm cells and sperm production [3]. Increased body temperature due to HS can affect mitochondrial activity, which increases the apoptosis rate in testicular germ cells leading to adverse effects on the spermatogenesis process [5]. In the present study, the phytochemicals extract treatment might be related to the decrease in ROS production under HS, which leads to a reduction in the incidence of apoptosis rate in germ cells. The antioxidant activity of phytochemicals extract (Table 2) may protect the germ cell in testes from apoptosis, leading to maintaining spermatogenesis and increasing sperm production [49]. The enhancement in relative epididymis weight of treatment groups might increase androgen secretion compared with the control [50]. Androgens, especially testosterone is necessary in the spermatogenesis process and their maintenance in the testis [51].

In the present study, the enhancement in semen quality observed could be related to the enrichment of turmeric and garlic extract with fatty acids, minerals, and vitamin components (Table 2). These compounds are efficient for semen production maintenance [49]. Dietary fatty acid manipulation was reported to affect the fatty acids profile of sperm plasma membrane and alter characteristics and function of spermatozoa [49,52]. The positive effect of treatment on the quality of semen may be about its role as a precursor of vitamin C, as an anti-stressor. Generally, several reports indicated that natural antioxidant activity could increase characteristics of heat-stressed rabbit semen [2,4,53].

In our study, the reduction observed in the concentration of total cholesterol and triglycerides was associated with a significant decrease in the relative weight of abdominal fat as affected by phytochemicals extract, which may be with a positive effect of garlic on lipid metabolism [14]. Also, dietary turmeric increases the 3-hydroxy-3-methylglutaryl coenzyme reductase inhibitor activity in rabbits [54]; consequently reduced 3-hydroxy 3-methylglutaryl coenzyme reductase leading to a marked reduction in the biosynthesis of total cholesterol in rat cells [55].

## Conclusion

Phytochemicals extract has a unique combination of phytochemicals. This product can be used as a promising new dietary additive supporting the future reproduction of rabbits under HS. These extracts could enhance heat tolerance, semen characteristics, and health status of rabbit males, particularly when used garlic extract at 100 mg per kg diet.

## List of Abbreviations

APRI, Animal Production Research Institute; CD, Commercial diet; ET, Ear temperature; HS, Heat stress; Hb, Hemoglobin; MDA, Malondialdehyde; PCV, Packed cell volume; PUFA, Poly-unsaturated fatty acids; ROS, Reactive oxygen species; RBCs, Red blood cells; RR, Respiration rate; SFA, Saturated fatty acids; TBA, Thiobarbituric acid; TAC, Total antioxidant capacity; UFA, Unsaturated fatty acids; WBCs, White blood cells.
